# Bilateral Peri-Scapular and Gluteal Elastofibromas: A Report of an Incidental Finding During Oncologic Follow-Up Imaging

**DOI:** 10.7759/cureus.84614

**Published:** 2025-05-22

**Authors:** Abdallah Said Abdallah, Fatima Zahrae Laamrani, Youssef Omor, Rachida Latib, Sanae Amalik

**Affiliations:** 1 Department of Radiology, National Institute of Oncology, Ibn Sina University Hospital Center, Rabat, MAR; 2 Faculty of Medicine and Pharmacy of Rabat, University Mohammed V, Rabat, MAR

**Keywords:** bilateral, computed tomography (ct), concomitant, elastofibroma, gluteal region, incidental finding, magnetic resonance imaging (mri), oncologic imaging, peri-scapular region

## Abstract

Elastofibroma is a benign soft-tissue tumor, typically located in the deep subscapular region. While bilateral dorsal elastofibromas are relatively common, the occurrence of multiple lesions involving distinct anatomical regions, particularly simultaneous involvement of the peri-scapular and gluteal areas, remains exceptionally rare. We report the case of a 64-year-old female patient undergoing oncologic surveillance for metastatic colorectal adenocarcinoma. During follow-up computed tomography (CT), incidental bilateral soft-tissue masses were identified adjacent to the serratus anterior muscles (peri-scapular) and within the gluteus medius muscles (gluteal regions). These lesions were asymptomatic and exhibited imaging features characteristic of elastofibromas, allowing for diagnosis without histologic confirmation. Stability of both gluteal and peri-scapular lesions was demonstrated on imaging follow-up. No specific treatment was required for these benign findings. This case highlights the exceptional rarity of concomitant bilateral elastofibromas involving both the peri-scapular and gluteal regions, particularly when incidentally discovered during oncologic follow-up. Vigilant radiologic assessment, with recognition of characteristic features even in atypical distributions, is essential to avoid misdiagnosis and unnecessary interventions, particularly in complex clinical settings.

## Introduction

Elastofibroma (EF) is a rare benign soft-tissue lesion, first described by Järvi and Saxén in 1961 [1]. Histologically, it is characterized by the proliferation of abnormal elastic fibers within a stroma of mature collagen and adipose tissue [2]. Typically, EF occurs in the subscapular or periscapular region, deep to the posterior thoracic wall muscles, a location considered almost pathognomonic [1,2]. It preferentially affects elderly women, often after the fifth or sixth decade [2].

Although initially considered exceptional, the prevalence of EF appears underestimated, with computed tomography (CT) studies reporting prevalences ranging from 0.8% to 2.7% in older individuals [3,4], and autopsy series finding rates as high as 24% [2]. A significant proportion of these lesions are asymptomatic and discovered incidentally during imaging performed for other reasons [5], and they are often omitted from initial radiology reports [5]. Radiologically, EF typically presents as a soft-tissue mass, often exhibiting alternating fibrous and fatty tissue, which results in a characteristic lamellated or streaky appearance, particularly with interspersed signal similar to fat on both CT and MRI. However, definitive diagnosis based on imaging alone can sometimes be challenging, especially in atypical locations or when attempting to differentiate from other soft-tissue lesions, including malignancies, in complex clinical settings such as oncologic surveillance, underscoring the need for awareness of its features. While the subscapular location is classic [1,2], atypical locations (e.g., ischial, olecranon, gluteal) have been described [6-8]. Bilateral subscapular lesions are common (10-66%) [2,7], but the presence of synchronous multifocal lesions in distinct anatomical sites is extremely rare [7,8]. Cases combining subscapular and gluteal/pelvic lesions have been reported very recently [6-8], suggesting a potentially underrecognized association.

Herein, we present a case illustrating this specific rare pattern, discovered incidentally, and discuss its diagnostic and management implications in light of the current literature.

## Case presentation

A 64-year-old female patient with a history of hypertension and asthma, who has been followed for a right-sided colon adenocarcinoma diagnosed in May 2023, underwent a total colectomy with ileorectal anastomosis (pathological staging: pT3N1bMx). Following adjuvant chemotherapy with the XELOX (capecitabine plus oxaliplatin) regimen, the patient experienced an early relapse with hepatic and pulmonary metastases, requiring initiation of palliative chemotherapy.

**Figure 1:**
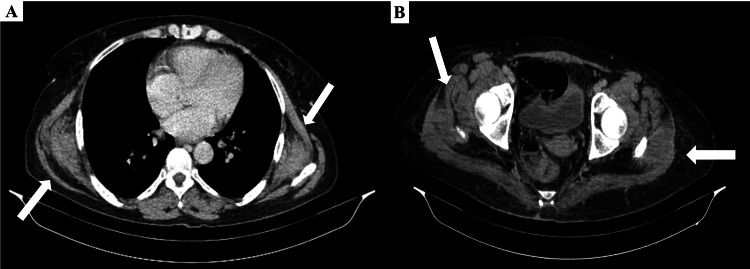
Contrast-enhanced CT (axial view) showing bilateral peri-scapular and gluteal elastofibromas on July 20, 2023 (A) Bilateral soft-tissue masses located deep to the serratus anterior muscles; (B) Bilateral soft-tissue lesions within the gluteus medius muscles(arrows). The lesions appear isodense relative to skeletal muscle and contain interspersed fatty streaks, suggestive of elastofibromas.

**Figure 2:**
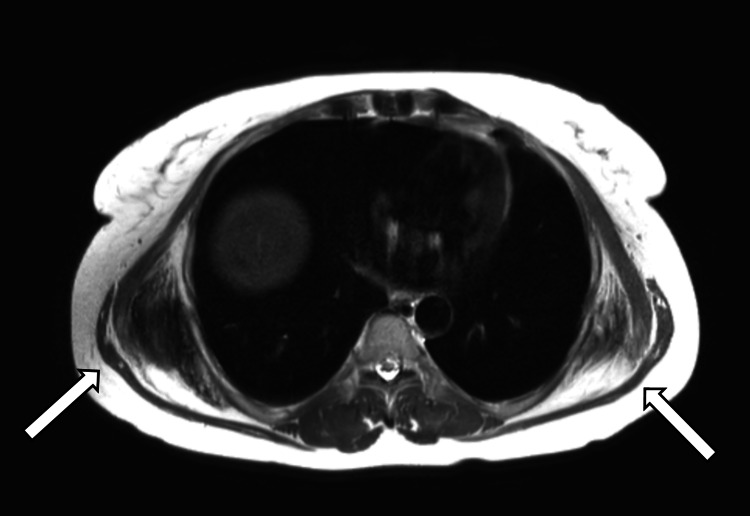
T2-weighted MRI sequence (axial view) without fat saturation on May 27, 2024 bilateral soft-tissue masses located deep to the serratus anterior muscles, displaying intermediate signal intensity with interspersed linear hyperintensities corresponding to internal fat strands (arrows), consistent with elastofibromas.

**Figure 3:**
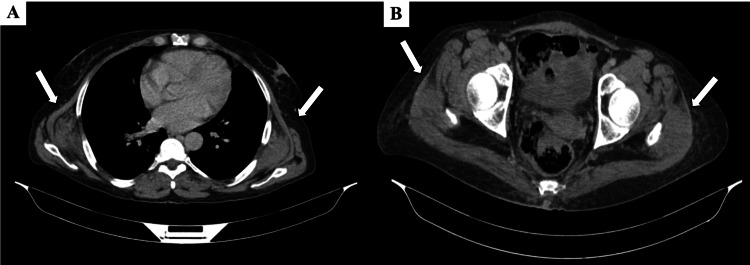
Follow-up contrast-enhanced CT (axial view) showing stability of bilateral elastofibromas on March 19, 2025 (A) Bilateral soft-tissue masses located in the peri-scapular region; (B) Bilateral lesions within the gluteus medius muscles. The lesions demonstrate a stable appearance compared to the initial scan performed in July 2023.

During oncologic follow-up, CT scans and magnetic resonance imaging (MRI) revealed incidental findings consistent with multiple bilateral EFs (Figures 1-3). The lesions showed a soft-tissue density similar to that of adjacent skeletal muscle and presented a stratified architecture with interspersed fatty streaks. There was no evidence of osseous involvement or infiltrative features.

The initial thoraco-abdomino-pelvic (TAP) CT scan performed on July 20, 2023, showed bilateral soft-tissue masses located at the thoracic wall, deep to the serratus anterior muscles, and within the gluteus medius muscles. Thoracic lesions measured 39 × 88 × 95 mm on the right and 32 × 80 × 97 mm on the left. Pelvic lesions measured 23 × 58 × 66 mm on the right and 24 × 56 × 61 mm on the left. Follow-up TAP CT performed on March 19, 2025, showed no significant change in size or morphology of these lesions compared to the initial scan. Radiology reports primarily focused on the metastatic disease. The patient was asymptomatic concerning these specific masses (no reported back or gluteal pain, snapping, or functional limitation). Given the characteristic bilateral distribution (peri-scapular and gluteal), the imaging appearance, and the asymptomatic status regarding these findings, a diagnosis of concomitant bilateral EF was established based on imaging alone. No biopsy or specific treatment was undertaken, and the lesions were placed under routine radiologic surveillance as part of the oncologic follow-up.

## Discussion

This case highlights an exceptionally rare pattern of EF distribution: simultaneous and bilateral involvement of both the peri-scapular region (deep to the serratus anterior) and the gluteal region (within the gluteus medius). EF typically affects women over 50-60 years of age [2], a profile consistent with that of our patient. While the subscapular location is by far the most common [1,2], atypical sites such as the gluteal region, although rare, are well-documented [6-8]. The simultaneous and bilateral occurrence in multiple sites, particularly scapular and gluteal, is exceptionally reported. Dandan et al. described a similar case in a 63-year-old man [7]. Indeed, synchronous pelvic or gluteal lesions associated with scapular EF, although very rare, are increasingly identified in the literature [6-8], potentially suggesting diagnostic underestimation in certain patient populations. Our case exemplifies this rare multifocal presentation. Ngoy et al. also described a case with involvement of four distinct sites [9]. This synchronous and multifocal presentation raises questions about the pathogenesis of EF. While the repetitive mechanical friction theory is often advanced to explain the subscapular location [1], it struggles to account for atypical or synchronous and multifocal forms [10]. Systemic factors, genetic predisposition [2], or an intrinsic disorder of elastic fiber formation [10] could instead be involved.

Incidental diagnosis, particularly during oncological follow-up utilizing frequent cross-sectional imaging, is becoming increasingly common. It is imperative for radiologists and clinicians to recognize the typical appearance of EF to avoid any misinterpretation, especially confusion with malignant or metastatic lesions in this context. Yanarateş et al. emphasized that such findings are frequently underreported. In our case, the diagnosis was confidently established based on imaging alone, thus obviating the need for biopsy [5]. Characteristic findings on CT and MRI, presenting as soft-tissue masses isodense or isointense to muscle with interspersed fatty streaks, are considered highly suggestive, particularly when bilateral [11].

The main differential diagnoses include deep lipomas and also malignant tumors such as liposarcomas or other soft tissue sarcomas [12], which are naturally of primary concern in the context of oncologic follow-up, as in our patient. Angiomatoid fibrous histiocytoma can also constitute a rarer differential diagnosis [13]. Furthermore, intramuscular myxomas can be considered in the differential for benign soft-tissue masses; however, these lesions typically exhibit distinct imaging features, such as a marked homogeneous high signal intensity on T2-weighted images due to their high fluid content, and they generally lack the characteristic interspersed fatty streaks evident in EFs [14]. The typical imaging appearance of EF, as demonstrated in our case with its pathognomonic interspersed fatty streaks and signal characteristics similar to muscle, generally allows for a confident distinction from these other entities, often obviating the need for biopsy, especially when stability is documented [11,12].

Given the complete absence of symptoms related to these lesions in our patient, a conservative approach based on radiological surveillance was favored. This strategy aligns with current recommendations for asymptomatic or minimally symptomatic EF [15,16], justified by their benign nature, the absence of reported malignant transformation [15], and the significant risk of postoperative complications (seroma, hematoma) associated with surgical excision [16]. The stability of the lesions observed over nearly 20 months of follow-up reinforces the relevance of this management. Surgical excision is indicated only in cases of significant symptoms [15,16].

## Conclusions

EF dorsi is a benign entity whose recognition is improving with the increasing use of cross-sectional imaging. We report an exceptional case of multiple, synchronous, bilateral EFs involving both the periscapular and gluteal regions, discovered incidentally in a patient undergoing oncologic follow-up. This case underscores the importance for clinicians and radiologists to be familiar with the typical imaging features of EF, which often permit a confident diagnosis without biopsy, even in atypical locations or multifocal presentations. It also illustrates the appropriateness of conservative management through surveillance for asymptomatic patients, considering the lesion's benignity and the risks associated with surgery. The observed multifocality may challenge purely mechanical pathogenetic theories and suggests the potential involvement of systemic or genetic factors.
